# Black Cumin Pressing Waste Material as a Functional Additive for Starch Bread

**DOI:** 10.3390/ma14164560

**Published:** 2021-08-13

**Authors:** Renata Różyło, Jolanta Piekut, Monika Wójcik, Katarzyna Kozłowicz, Marzena Smolewska, Marta Krajewska, Marek Szmigielski, Hayat Bourekoua

**Affiliations:** 1 Department of Food Engineering and Machines, University of Life Sciences in Lublin, 28 Głęboka Str., 20-612 Lublin, Poland; monika.wojcik@up.lublin.pl; 2 Department of Agricultural, Food and Forestry Engineering, Bialystok University of Technology, 45E Wiejska Str., 15-351 Białystok, Poland; j.piekut@pb.edu.pl; 3 Department of Biological Bases of Food and Feed Technologies, University of Life Sciences in Lublin, 28 Głęboka Str., 20-612 Lublin, Poland; katarzyna.kozlowicz@up.lublin.pl (K.K.); marta.krajewska@up.lublin.pl (M.K.); marek.szmigielski@up.lublin.pl (M.S.); 4 Faculty Chemical Laboratory, Bialystok University of Technology, 45E Wiejska Str., 15-351 Białystok, Poland; m.smolewska@pb.edu.pl; 5 Laboratoire de Nutrition et Technologie Alimentaire (LNTA), Institut de la Nutrition, de l’Alimentation et des Technologies Agro-Alimentaires (INATAA), Equipe de Transformation et Elaboration de Produits Agro-Alimentaires (TEPA), Université Frères Mentouri-Constantine 1, Route de Ain El-Bey, Constantine 25000, Algeria; bourekoua.h@hotmail.fr

**Keywords:** black cumin, *Nigella sativa*, phenolic compounds, bread, waste materials

## Abstract

The aim of the study was to determine the effect of the addition of black cumin (*Nigella sativa* L.) pressing waste (BCW) and black cumin seeds (BCS) on the properties of starch bread. The control bread was prepared from wheat starch (100%) with a gluten-free certificate, plantain husk (5%), and guar gum (2%). BCS and BCW were added between 0 and 10% of wheat starch. We determined the physicochemical properties, color, texture, and sensory properties of the prepared bread. Gas chromatography–mass spectrometry (GC–MS) analysis was performed to detect the phenolic compounds in the bread. The bread prepared with 6% BCS and 4% BCW had a significantly higher volume than the starch control bread did. Sensory analysis (taste) showed that BCS and BCW could be added up to 4% and 8%, respectively. The addition of BCS and BCW reduced the brightness of the crumb. A significant decrease in the L * index of the crumb was observed from 50.9 for the control bread to 34.1 and 34.0 for bread with 10% BCS and BCW, respectively. The addition of BCS and BCW decreased the hardness, elasticity, and chewiness of the starch bread crumb. Starch bread enriched with BCS and BCW was characterized by a higher content of 2-hydroxybenzoic acid, 2-hydroxyphenyl acetic acid, and 4-hydroxyphenyl acetic acid.

## 1. Introduction

Bread is one of the most popular food products around the world. Most people today suffer from various forms of allergies and health conditions; therefore, it is necessary to develop adequate dietary products. Carbohydrates, mainly starch from cereals, play an important part in our diet [1,2], and according to the dietary guidelines, a diet with a low glycemic index, e.g., rich in slowly digestible carbohydrates, is important [3]. Protein from traditional cereals is often intolerant to some groups of consumers, such as those suffering from celiac disease [4,5] and phenylketonuria [6]. Additionally, it has to be mentioned that people with phenylketonuria must be careful about taking Phe from any sources. Alternative bread baking recipes for such people are being searched for.

In this study, we proposed the enrichment of starch bread with black cumin pressing waste (BCW). Previous research has shown that waste generated from various food materials has a high nutritional value and is rich in dietary fiber, minerals, and antioxidants [7].

Researchers have attempted to enrich bakery products with apple pomace, buckwheat pomace, grape pomace [8], carrot pomace [9], pitaya peel powder [10], and lettuce waste flour [11]. In our previous studies, we demonstrated the usefulness of chia pressing waste in the production of bread [12].

In this study, we aimed to utilize BCW in the making of bread. Black cumin (BC) (*Nigella sativa* L.) has been used as a traditional medicine for centuries [13,14,15]. BC contains many active components, including thymoquinone (TQ), thymohydroquinone, dithymoquinone, thymol, carvacrol, nigellimine-N-oxide, nigellicine, nigellidine, and alpha-hederin [16]. TQ, the principal active constituent of BCS, exhibits various properties, including anticancer and chemosensitizing properties [17]. The oilseed from BCS shows antioxidant and anti-cancer properties [18]. Black cumin can be used as a potential therapeutic agent for asthma [19]. The literature shows that BCS significantly improves the parameters of hyperglycemia and controls diabetes [20,21]. Moreover, BCS shows other pharmacological effects such as immune stimulation and the reduction in arterial hypertension [16].

Previously, studies have been conducted on the addition of BCS to wheat bread [22,23] or gluten-free bread [24]. Another study showed the use of BCS protein concentrate for baking gluten-free bread [25]. However, there is no information on the use of BCS in starch bread [26].

So far, no comprehensive research has been carried out on the properties and applications of BCW in the recipe of starch bread. Therefore, in this study, we tested various starch bread recipes prepared with the addition of BCS and BCW. To the best of our knowledge, this is the first study to describe the GC–MS detection of phenolic compounds in an innovative BCW-enriched starch bread. These compounds were also detected for the first time in BCS and BCW.

## 2. Materials and Methods

### 2.1. Materials

Gluten-free wheat starch (Glutenex, Sady, Poland) and Plantago ovata husk (Targroch, Filipowice, Poland) were used as the raw materials for the production of control bread. Black cumin seeds were sourced from India (Targroch, Filipowice, Poland). The same batch of seeds was used for BCS and BCW. Other materials used in this study were guar gum, dried instant yeast (Instaferm, Lallemand Iberia, Setubal, Portugal), and salt.

### 2.2. Chemical Composition of Raw Materials and Calorific Value of Bread

The chemical composition of wheat starch, BCS, BCW, and bread, such as protein content, was determined by the Kjeldahl method (Kjeltec 2300, Foss) [27], fat by the Soxhlet method (Soxtec 2050, Foss) [28], ash by incineration [29], moisture content by the drying method [30], and the dietary fiber content according to the method of Asp et al. [31]. The amount of carbohydrate was calculated by subtracting the protein, fat, moisture, and dietary fiber content. The calorific value (per 100 g of bread) was calculated according to Costantini et al. [32] using the Atwater coefficients. The fatty acid composition was determined by gas chromatography (GC) (Bruker 436GC chromatography with FID detector, Billerica, MA, USA) according to appropriate standards [33]. The fatty acid methyl esters were separated on a BPX 70 capillary column (60 m × 0.25 mm, 25 µm, Trajan Scientific and Medical, Melbourne, Australia) with nitrogen as the carrier gas. All measurements were made in triplicate.

### 2.3. Black Cumin Seed Waste Pressing

The seed waste was obtained as a by-product of the cold pressing of N. sativa seeds using a DUO-type screw press (Farmet, Česká Skalice, Czech Republic). Both raw materials were ground in a knife grinder before baking.

### 2.4. Process of Starch Bread Baking

Starch bread was baked using a single-phase method [34]. The control starch bread was baked from gluten-free wheat starch (100%) and the recipe was supplemented with p. ovata husk (5%), guar gum (2%), dried instant yeast (1%), and salt (2%). The amount of water addition was 130%, which was determined experimentally [35]. The main ingredients for the production of the control bread were gluten-free wheat starch and p. ovata husk. It has been proven that it is impossible to make bread from starch alone, so additives are needed to improve the quality of the bread [36,37,38]. Our primary goal was to create the least complicated recipe based on natural additives, as more and more consumers have been paying attention to clean labels. The starch bread recipe was developed by testing the addition of varying amounts of p. ovata husk ranging from 1% to 6% to wheat starch. The bread made of wheat starch alone was very hard and crumbled. The increasing proportion of p. ovata husk significantly improved the parameters of the bread, including the volume and texture of the crumb. However, there were no significant differences between the bread with 5% and the 6% p. ovata husk; therefore, the 5% supplement was considered optimal. Other studies have confirmed that the consistency of the dough was influenced by the addition of dietary fiber due to the hydration properties of the fibers [39]. Despite the significant improvement, the uneven porosity of the softener was observed; therefore, we used 2% guar gum, which had a positive effect on the volume, texture, and porosity of the bread. Guar gum is a natural ingredient often used in gluten-free bread recipes [40,41].

The control bread recipe was enriched with the addition of N. sativa seeds (0%, 2%, 4%, 6%, 8%, and 10%) and waste material obtained after pressing the oil from these seeds (0%, 2%, 4%, 6%, 8%, and 10%).

The dough was mixed for 5 min (Kitchen Aid, St. Joseph, MI, USA), and then divided into 300 g portions, which were fermented and proved in molds in a fermentation chamber (Sadkiewicz Instruments, Bydgoszcz, Polska) (30 °C, 75% RH, 40 min), and then baked in a laboratory oven (Sadkiewicz Instruments, Bydgoszcz, Polska) (220 °C, 40 min). After baking and cooling, the loaves were packed into polyethylene bags and analyzed after 24 h. Baking tests were performed in triplicate and used for physical and chemical analysis. An additional 5 loaves were made from each sample for sensory analysis.

### 2.5. Determination of Basic Physical Parameters of Bread

The bread volume was measured by using the millet seeds displacement method, and then the specific volume of bread (bread volume divided by weight) was calculated. The pH value of the breadcrumb was determined using a pH meter (TESTO 206-ph2, Pruszków, Poland). Color measurements were performed on the L *a * b * scale (4Wave CR30-16) (Planeta, Tychy, Poland), and ΔE was calculated [42,43]. Measurements of these physical parameters of bread were performed in 3 replications.

### 2.6. Determination of Texture and Sensory Parameters of Bread

The texture parameters of breadcrumbs (30 mm × 30 mm × 20 mm) were determined in a double compression test to a depth of 50% with a speed of 1 mm∙s^−1^ (ZWICK Z020/TN2S, ZwickRoell, Ulm, Germany). The hardness, springiness, cohesiveness, and chewiness were calculated from the graphs obtained during the TPA test (texture profile analysis). The texture measurements were performed in 9 replicates in the middle of the central slices of the bread.

The sensory analysis was performed by a panel of 75 untrained consumers (21–55 years old), who rated the taste, aroma, appearance, texture, and overall acceptability of the bread samples. The responses were obtained with the use of a 9-point hedonic scale (9—Extremely like, 8—Very much like, 7—Quite like, 6—Moderately like, 5—Neither like or dislike, 4—Moderately dislike, 3—Quite dislike, 2—Very much dislike, and 1—Extremely dislike) [44].

### 2.7. Extraction and Derivatization of Phenolic Compounds

The 5 g powdered seeds, waste material, and dried bread were extracted thrice with 40 mL of 80% acidified methanol in 40 °C with sonification. In the next step, the supernatant was evaporated under reduced pressure to remove all methanol. Aqueous fractions were extracted with *n*-hexane to remove the lipid fraction [45,46].

Phenolic compounds were extracted with 2 × 10 mL portions of diethyl ether/ethyl acetate (*v*/*v* 1:1). The collected eluent was dried over anhydrous sodium sulfate and then evaporated to dryness on a rotary evaporator under vacuum. The extracted dry residue was derivatized with 100 µL of N,O-bis(trimethylsilyl) trifluoro acetamide (BSTFA) with 1% trimethyl chlorosilane (TMCS) (for GC derivatization, Supelco, Bellefonte, PA, USA) and 200 µL of pyridine (anhydrous, 99.8%, Sigma-Aldrich, St. Louis, MO, USA). The content was heated at 60 °C for 1 h. Trimethylsilyl (TMS) derivatives were subjected to GC-MS analysis.

### 2.8. C-MS Analysis

The separation and detection of phenolic compounds were conducted using a 7890B GC System with a 7000C GC/MS Triple Quad mass detector (Agilent Technologies, Santa Clara, CA, USA). The determination of phenolic acids was made based on calibration curves of individual standard compounds. The identification was carried out based on the mass spectra and the chromatogram of a standard mixture of 30 different phenolic acids analyzed under the same conditions of the chromatographic procedure. A HP-5 ms fused silica capillary column (30 m × 0.25 mm × 0.25 μm, Agilent Technologies) was used for the separation process. The injection temperature was maintained at 260 °C, and the carrier gas flow rate was maintained at 1 mL∙min^−1^ (helium). The chromatographic analysis was based on a validated procedure [47]. Temperatures from 40 to 300 °C were programmed at a rate of 3 °C·min^−1^ (1:10 split) to separate compounds. The analyses were carried out in full scan mode, and we wanted the best possible separation of the mixture of analytes, some of which are isomers, with slightly different retention indices, while others had a significant molecular weight and retention rates above 3000. The detection process was performed in the full scan mode from 45 to 600 m/z. All compounds were calibrated using the same parameters.

### 2.9. Statistical Analysis

Statistical analysis was conducted using Statistica software version 12.0 considering a significance level of α = 0.05. Analysis of variance (ANOVA) was performed, and Tukey’s test was used to compare the mean values.

## 3. Results and Discussion

### 3.1. Chemical Composition of Starch and BCS and BCW

The bread was supplemented with BCS or BCW. The composition of the BCS was as follows: protein, 20.0 ± 0.8%; fat, 34.6 ± 1.3%; fiber, 12.6 ± 0.9%; and carbohydrates, 26.3 ± 0.8%. The composition of the BCW was as follows: protein, 26.0 ± 0.6%; fat, 18.3 ± 0.5%; fiber, 16.3 ± 1.5%; and carbohydrates, 34.1 ± 2.1%.

Among the fatty acids detected (Table 1) in BCS and BCW, the highest amount (18.95 g/100 g; 10.02 g/100 g) was of C18:2 linoleic (n − 6) acid, the second-highest amount (8.39 g/100 g; 4.43 g/100 g) was of C18:2 oleic (n − 9) acid, and the third (4.52 g/100 g; 2.39 g/100 g) was of C16:0 palmitic acid. Stearic acid (C18:0) was detected in an amount equal to 1.45 g/100 g in BCS and 0.77 g/100 g in BCW. The amount of eicosadienic acid (C20:2) was 0.87 g/100 g in BCS and 0.46 g/100 g in BCW of 0.36 ± 0.03, C18:3 α-linolenic (n − 3) acid of 0.28 ± 0.03, C 16:1 palmitoleic of 0.21 ± 0.02, C20:0 arachidic acid of 0.18 ± 0.01, C12:0 lauric acid of 0.14 ± 0.01. Other authors’ findings have also shown a high proportion of linoleic, oleic, and palmitic acids. A large proportion of petroselinic acid has also been detected [48].

### 3.2. Physical Properties and Color Values of Starch Bread with BCS and BCW

The results showed that the starch bread with the addition of BCS and BCW increased its specific volume (Table 2). The bread prepared with 6% BCS and 4% BCW had a significantly larger volume than the starch control bread did. There were no significant differences between the volume of bread with the addition of 4%, 6%, 8%, and 10% BCS content and the 2%, 4%, 6%, 8%, and 10% BCW content. The porous structure of the bread is due to the ability to retain fermentation gases, which increases the volume. The volume of the starch bread can be improved by adding protein, which initially absorbs water and swells together with the gelatinizing starch granules to form a dough structure [49]. According to a previous study, the BCS protein concentrate increased the water content in the reduced loaf volume [25]. In this study, we used optimal quantities of water, and the recipe of control starch bread was based on natural additives such as guar gum and plantain husk, which did not contain fat. Therefore, the addition of BCS and BCW, which also had a low fat content, could have a positive effect on the volume of bread. According to other studies, oil additives to bread dough act as surfactants that can bind to starch granules, thereby stabilizing and strengthening the dough, which could consequently increase the volume of bread [23,48].

A significant decrease in the pH value was observed (Table 2) after the addition of BCS from 6.09 for the control bread (C) to 5.52 for bread with 6% BCS. However, the addition of 4% BCW resulted in a significant decrease in the pH value from 6.09 for the control bread to 5.49 for the bread with BCW. The pH values were not significantly different for each proportion of both BCS and BCW.

The addition of BCS and BCW reduced the brightness of the crumb (Table 2), which was caused by the dark color of the BCS. No major differences were observed between BCS and BCW for the same levels of additive. The increasing proportion of BCS and BCW caused a significant reduction in the value of the L * index from 50.9 for the control bread to 34.1 and 34.0 for bread with a 10% BCS and BCW, respectively. Similar to the L * values, the a * and b * values changed with the increase in the proportions of BCS and BCW, respectively. This affected the ΔE parameter, which changed from 9.93 or 6.97, respectively, for 2% BCS or BCW to 17.20 or 16.85, respectively, for 10% BCS or BCW. So far, there are no studies on the effect of the addition of BCW on the quality of starch bread, but a similar relationship regarding the color of the crumb was observed in other studies in which defatted BCS flour affected the darkening of traditional wheat bread [22]. Considering color, gluten-free bread samples with BCS protein concentrate were distinctly darker [25]. Similarly, in an earlier study, the addition of chia pressing waste caused a significant darkening of the bread crumb [12].

### 3.3. Texture and Sensory Evaluation of Starch Bread with BCS and BCW

**Figure 1:**
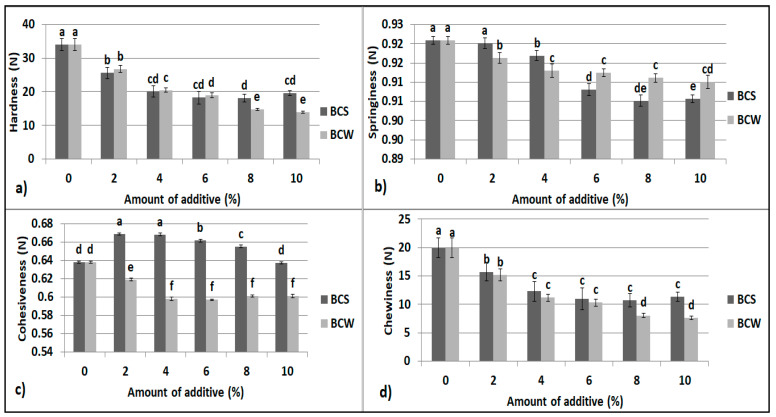
Textural properties of starch bread with BCS and BCW: (**a**) hardness, (**b**) springiness, (**c**) cohesiveness, (**d**) chewiness; mean values in the same figure marked with different letters are significantly different (α = 0.05).

The addition of BCS and BCW decreased the hardness, elasticity, and chewiness of the starch bread crumb (Figure 1a–d). Only crumb cohesiveness increased in the case of the addition of BCS in the range of 2%–8%. The addition of BCW reduced the cohesiveness. With a smaller proportion ranging from 2% to 4%, the springiness was higher for BCS than for BCW, whereas, with the proportion ranging from 8% to 10%, the springiness was significantly higher for BCW than for BCS.

In a previous study, the BCS protein concentrate significantly increased the firmness and decreased the springiness of the crumb of gluten-free bread [25]. The oil of BCS tested as an additive to gluten-free bread resulted in significantly softer breadcrumbs [23]. A similar relationship has been noted in our study. Gluten-free bread tends to have a worse texture, including greater hardness and lower springiness than traditional bread [12,50,51]. The reduction in crumb hardness in gluten-free starch bread may be due to the enrichment of the dough with natural emulsifiers. Moreover, the addition of structure-forming substances, for example, proteins, strengthens the crumb, improves flexibility, and improves the retention of fermentation gases [49].

**Figure 2:**
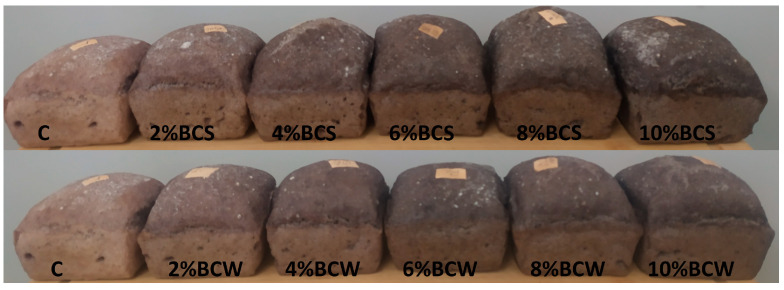
The external appearance of bread with different amounts of BCS and BCW: C—control starch bread, BCS—black cumin, BCW—black cumin pressing waste.

**Figure 3:**
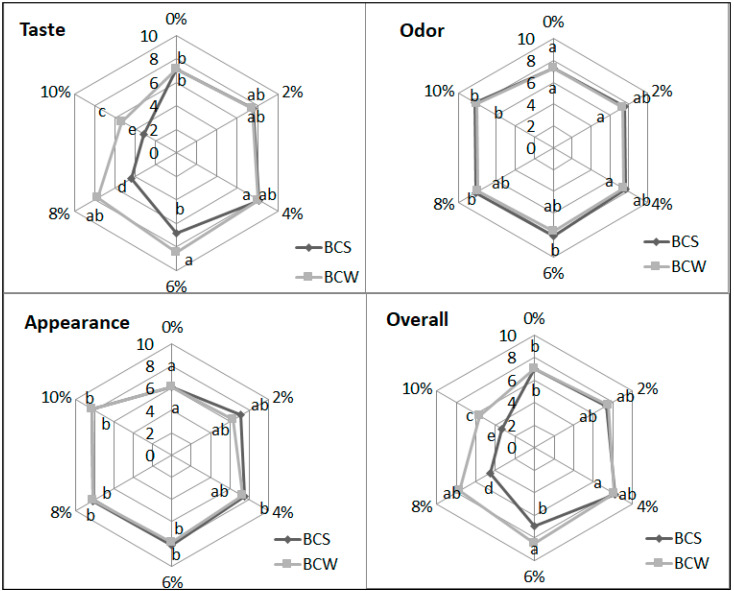
Sensory evaluation of starch bread with BCS and BCW: C—control starch bread, BCS—black cumin, BCW—black cumin pressing waste; mean values in the same figure marked with different letters are significantly (α = 0.05) different.

Sensory analysis (Figure 2 and Figure 3) of the starch bread showed that the best taste of the bread was rated with 6% BCW. Some consumers tolerated 6% BCS, although it provided a bitter taste when swallowed. However, according to the obtained responses, BCW was tolerated up to a quantity of 6% and even 8%. The smell of bread was better judged for samples with a higher proportion of BCS and BCW. The appearance of the bread and texture with both BCS and BCW was judged as better for a higher proportion of additive. The overall rating showed optimal 4% BCS and 6–8% BCW as the optimum proportions. In another study, the overall acceptability of the wheat bread with defatted BCS flour was significantly lower than in the control, where 10% and 15% showed similar acceptability values, while 5% was closer to the control sample [22]. Research has shown that BCS oil contains volatile compounds, which also affect the taste and aroma of bread [47]. BCS containing more fat probably had more aromatic compounds; therefore, bread with BCW was more tolerated than with BCS.

### 3.4. Calorific Value of Starch Bread with BCS and BCW

The control starch bread had the following characteristics: protein, 0.15%; fat, 0.10%; fiber, 2%; and carbohydrate, 44.9%. The addition of BCW compared to BCS increased the levels of protein and fiber in the bread (Table 3), while the fat content was increased in the bread prepared with BCS. Increasing the fat content of the bread from the addition of BCS appears to be nutritionally beneficial. As mentioned in the previous section, linoleic (n − 6) acid, oleic (n − 9) acid, palmitic acid, stearic acid, eicosadienic acid, eicosenoic acid, α-linolenic (n − 3) acid, palmitoleic acid, arachidic acid, and lauric acid were detected in BCS and BCW.

Control starch bread had a low caloric value of 185.2 kcal/100 g (Table 3). This might be because the bread was made from wheat starch alone with 5% plantain husk and 2% guar gum added to it. Usually, bread has a caloric value higher than 200 kcal/100 g [12,32]. Bread with an optimal addition of BCS (4%) was characterized by a caloric value of 188.9 kcal/100 g, and bread with 6% BCW was characterized by a caloric value of 188 kcal/100 g. Bread with 8% BCW had a caloric value of 200.3 kcal/100 g.

### 3.5. Phenolic Acid Content in Starch Bread with BCS and BCW Detected by GC-MS Analysis

Table 4 presents the phenolic acid content detected by GC-MS analysis in starch bread with BCS and BCW.

The analysis of phenolic compounds showed that the enrichment of starch bread with BCS and BCW resulted in a significant increase in the content of 2-hydroxybenzoic acid (salicylic acid). Salicylic acid levels were increased with the addition of 4% BCS. Its content was 1.87 times more than in BCS bread than that of control bread. The addition of 4% BCW resulted in 3.6 times increase in the level of salicylic acid when compared to the control bread. In contrast, salicylic acid in bread with 8% BCW was 5.4 times than that of the control bread. In starch control bread, 2-hydroxybenzoic acid was 0.2004 μg/g d.m, and in bread with 4% BCS and 8% BCW, it was, respectively, 0.3767 μg/g d.m and 1.0903 μg/g d.m.

Recent data indicate that in addition to antioxidant properties, hydroxybenzoic acids are capable of inhibiting α-amylase and α-glucosidase, enzymes that break down complex carbohydrates by keeping blood sugar levels low [51]. The increasing addition of BCS and BCW resulted in proportional increases in 2-hydroxyphenylacetic acid and 4-hydroxyphenylacetic acid. The control bread had a cinnamic acid content of 1.4404 μg/g d.m. and the bread with 4% BCS had 1.5073 μg/g d.m. Wheat bread with 8% BCW had a cinnamic acid content of 1.4626 μg/g d.m. In the case of 2-methoxybenzoic acid (anisic acid), only 6% or 8% of BCW additive showed a positive effect on a significant increase in its content.

## 4. Conclusions

The obtained results confirm the possibility of the utilization of waste from black cumin pressing in the production of gluten-free starch bread. These wastes added to the bread recipe in an amount not exceeding 8% made the bread more interesting in taste and appearance. Moreover, such bread was beneficial in phenolic content. However, the higher amounts of BCW negatively affected the taste and odor of bread (unpleasant aroma and bitter taste). Bread with 4% BCS was characterized by a caloric value of 188.9 kcal/100 g and that of 8% BCW was characterized by a calorific value of 200 kcal/100 g. Among the fatty acids identified in BCS and BCW, the highest quantities were recorded for C18:2 linoleic (n − 6) acid, followed by C18:2 oleic (n − 9) acid, and C16:0 palmitic acid. The enrichment of starch bread with BCS and BCW resulted in a significant increase in the content of 2-hydroxybenzoic acid (salicylic acid), 2-hydroxyphenylacetic acid, and 4-hydroxyphenylacetic acid.

## Figures and Tables

**Table 1:** Composition of fatty acids in black cumin (BCS) seeds and black cumin pressing waste (BCW).

Fatty Acids	Black Cumin Seeds (BCS) (g/100 g) Mean ± SD *	Black Cumin Pressing Waste (BCW) (g/100 g) Mean ± SD *
C 12:0 lauric acid	0.048 ± 0.006 a	0.026 ± 0.004 b
C 14:0 myristic acid	0.006 ± 0.002 a	0.003 ± 0.001 b
C 16:0 palmitic acid	4.522 ± 0.156 a	2.392 ± 0.110 b
C 16:1 palmitoleic acid	0.074 ± 0.011 a	0.039 ± 0.005 b
C 18:0 stearic acid	1.453 ± 0.103 a	0.769 ± 0.043 b
C 18:1 oleic (n − 9) acid	8.389 ± 0.130 a	4.437 ± 0.112 b
C 18:2 linoleic (n − 6) acid	18.954 ± 0.244 a	10.025 ± 0.201 b
C 18:3 α-linolenic (n − 3) acid	0.096 ± 0.011 a	0.051 ± 0.003 b
C 20:0 arachidic acid	0.063 ± 0.004 a	0.034 ± 0.001 b
C 20:1 eicosenoic acid	0.123 ± 0.011 a	0.065 ± 0.004 b
C 20:2 eicosadienic acid	0.871 ± 0.090 a	0.461 ± 0.022 b

* Values in the same row marked with different letters are significantly (α = 0.05) different. Abbreviation: SD, standard deviation.

**Table 2:** Basic physical properties and crumb color values of bread.

Kind of Sample	Specific Volume (cm^3^/g)	pH-Value	Crumb Color Values			
			L *-Value	A *-Value	B *-Value	ΔE
C	1.56 ± 0.06 a	6.09 ± 0.31 a	50.9 ± 0.3 a	5.07 ± 0.04 a	7.62 ± 0.06 a	-
2% BCS	1.60 ± 0.07 a	5.75 ± 0.29 ab	41.1 ± 0.4 c	3.87 ± 0.12 b	6.90 ± 0.09 c	9.93
4% BCS	1.65 ± 0.07 a	5.68 ± 0.22 ab	39.2 ± 0.2 d	2.83 ± 0.06 d	6.40 ± 0.11 d	12.05
6% BCS	1.76 ± 0.07 b	5.52 ± 0.24 b	37.4 ± 0.3 e	2.31 ± 0.04 e	6.46 ± 0.03 d	13.86
8% BCS	1.74 ± 0.08 b	5.46 ± 0.21 b	35.8 ± 0.2 f	1.50 ± 0.02 h	6.82 ± 0.11 c	15.51
10% BCS	1.72 ± 0.07 b	5.31 ± 0.25 b	34.1 ± 0.2 g	1.41 ± 0.01 i	6.88 ± 0.09 c	17.20
2% BCW	1.70 ± 0.08 ab	5.67 ± 0.27 ab	44.1 ± 0.3 b	3.58 ± 0.01 c	6.98 ± 0.06 bc	6.97
4% BCW	1.78 ± 0.08 b	5.49 ± 0.25 b	39.8 ± 0.5 d	2.99 ± 0.05 d	7.26 ± 0.07 b	11.30
6% BCW	1.82 ± 0.07 b	5.39 ± 0.26 b	37.1 ± 0.4 e	2.11 ± 0.05 f	7.20 ± 0.09 b	14.15
8% BCW	1.79 ± 0.08 b	5.33 ± 0.29 b	35.6 ± 0.2 f	1.71 ± 0.01 g	7.63 ± 0.07 a	15.66
10% BCW	1.76 ± 0.09 b	5.25 ± 0.21 b	34.0 ± 0.3 g	1.45 ± 0.02 hi	7.52 ± 0.09 a	16.85

* mean values in the same column marked with different letters are significantly (α = 0.05) different.

**Table 3:** Chemical composition and calorific value of starch bread prepared with BCS and BCW.

Kind of Sample	Protein (%)	Fat (%)	Fiber (%)	Carbohydrates (%)	Calorific Value kcal/100 g
C	0.15 ± 0.005 a	0.10 ± 0.002 a	2.03 ± 0.17 a	44.91	185.2
2% BCS	0.37 ± 0.007 b	0.47 ± 0.003 c	2.16 ± 0.18 ab	44.26	187.1
4% BCS	0.58 ± 0.015 c	0.84 ± 0.026 e	2.29 ± 0.19 b	43.60	188.9
6% BCS	0.79 ± 0.022 d	1.21 ± 0.009 f	2.43 ± 0.16 bc	42.95	190.8
2% BCW	0.43 ± 0.006 e	0.30 ± 0.006 b	2.20 ± 0.17 ab	44.34	186.2
4% BCW	0.71 ± 0.012 f	0.50 ± 0.011 c	2.38 ± 0.18 bc	43.77	187.1
6% BCW	0.98 ± 0.018 g	0.68 ± 0.017 d	2.55 ± 0.19 c	43.20	188.0
8% BCW	1.27 ± 0.025 h	0.89 ± 0.023 e	2.73 ± 0.21 c	45.45	200.3

Mean values in the same column marked with different letters are significantly (α = 0.05) different. Abbreviations: C—control starch bread, BCS—black cumin, BCW—black cumin pressing waste.

**Table 4:** Phenolic acids detected by GC-MS analysis in starch bread with BCS and BCW.

µg/g d.m.	C	2% BCS	4% BCS	6% BCS	BCS	2% BCW	4% BCW	6% BCW	8% BCW	BCW
Phenoxyacetic acid	0.1163 d ± 0.0021	0.1029 c ± 0.0097	0.1044 c ± 0.0099	0.1031 c ± 0.0077	0.0230 a ± 0.0013 a	0.1098 cd ± 0.0098	0.1080 cd ± 0.0102	0.1146 cd ± 0.0113	0.1153 cd ± 0.0088	0.0774 b ± 0.0055 b
2-Methoxybenzoic acid (Anisic)	0.0048 abc ± 0.0013	0.0042 ab ± 0.0004	0.0047 bc ± 0.0005	0.0039 abc ± 0.0011	0.0039 a ± 0.0004	0.0046 abc ± 0.0012	0.0046 bc ± 0.0004	0.0051 c ± 0.0006	0.0051abc ± 0.0010	0.0043 abc ± 0.0013
2-Hydroxybenzoic acid (Salicylic)	0.2004 a ± 0.0017	0.3326 b ± 0.0086	0.3767 c ± 0.0303	0.5254 d ± 0.0029	7.7710 h ± 0.5983	0.6096 e ± 0.0553	0.7210 f ± 0.0284	0.9193 g ± 0.1040	1.0903 g ± 0.0992	9.5139 i ± 0.2078
Cinnamic acid	1.4404 c ± 0.0697	1.4516 c ± 0.1024	1.4919 c ± 0.0861	1.5073 c ± 0.2044	0.0771 a ± 0.0066	1.4475 c ± 0.1055	1.4480 c ± 0.0997	1.4593 c ± 0.1144	1.4626 c ± 0.1206	0.1098 b ± 0.0082
2-Hydroxyphenylacetic acid	0.2545 a ± 0.0203	0.2999 ab ± 0.0311	0.2996 b ± 0.0212	0.3422 c ± 0.0099	0.4510 de ± 0.0408	0.3750 d ± 0.0321	0.3837 d ± 0.0299	0.5146 e ± 0.0358	0.9769 f ± 0.0881	2.2954 g ± 0.2246
4-Hydroxyphenylacetic acid	0.7185 b ± 0.0351	0.7242 b ± 0.0460	0.7846 bc ± 0.0594	0.8355 c ± 0.0701	0.3552 a ± 0.0299	0.7521 b ± 0.0078	0.8564 c ± 0.0630	0.9255 c ± 0.1061	0.9649 c ± 0.1022	1.8789 d ± 0.1605
o-Coumaric acid	0.0097 d ± 0.0004	0.0071 b ± 0.0015	0.0088 c ± 0.0008	0.0095 cd ± 0.0008	0.0055 a ± 0.0011	0.0122 de ± 0.0021	0.0146 e ± 0.0009	0.0150 e ± 0.0012	0.0152 e ± 0.0011	0.0756 f ± 0.0063
p-Coumaric acid	0.0386 bc ± 0.0022	0.0410 bc ± 0.0042	0.0419 bc ± 0.0043	0.0490 d ± 0.0024	0.0218 a ± 0.0018	0.0376 bc ± 0.0032	0.0385 b ± 0.0012	0.0425 c ± 0.0029	0.0455 cd ± 0.0028	0.0425 bcd ± 0.0054
Ferulic acid	-	-	-	-	0.0051 a ± 0.0007	-	-	-	0.0046 b ± 0.0012	0.0395 c ± 0.0064
Sinapinic acid	-	-	-	-	-	-	-	-	-	0.0015 a ± 0.0006
Chlorogenic acid	-	-	-	0.0784 a ± 0.0072	0.4241 b ± 0.0398	-	-	-	0.1113 a ± 0.0431	0.5561 b ± 0.1588

C—control starch bread, BCS—black cumin seeds, BCW—black cumin pressing waste. Mean values in the same row marked with different letters are significantly (α = 0.05) different.

## Data Availability

Correspondence and requests for materials should be addressed to R.R.
